# Effect of hydration on urine and serum *Histoplasma* antigen levels in patients with disseminated histoplasmosis

**DOI:** 10.1128/spectrum.03714-23

**Published:** 2024-04-12

**Authors:** Michael Dzombo, Allyson L. Hughes, Sara L. Hollis, Christopher H. Trabue, George E. Nelson, Richard W. Larue, Molly Busenbark, L. Joseph Wheat, Melissa L. Scalise

**Affiliations:** 1 Department of Medicine, Ascension/St. Thomas, University of Tennessee Health Science Center, Nashville, Tennessee, USA; 2 Department of Medicine, Division of Infectious Disease, Vanderbilt University Medical Center, Nashville, Tennessee, USA; 3 MiraVista Diagnostics and MiraBella Technologies, Indianapolis, Indiana, USA; Mayo Foundation for Medical Education and Research, Rochester, Minnesota, USA

**Keywords:** *Histoplasma*, disseminated histoplasmosis, *Histoplasma* antigen, hydration status

## LETTER

*Histoplasma* antigen detection assays have emerged as common tools for diagnosing disseminated histoplasmosis. Urine *Histoplasma* antigen (UHAg) is highly sensitive; however, variability has been noted and hypothesized to be related to the effects of hydration on antigenuria in previous studies (1–4). This study describes changes in UHAg and serum *Histoplasma* antigen (SHAg) levels associated with hydration in patients with disseminated histoplasmosis.

Patients with a new diagnosis of disseminated histoplasmosis were recruited from the Middle Tennessee area from October 2020 to October 2022. Patients diagnosed within the previous 6 months had positive histoplasmosis culture or antigen testing, age ≥ 18 years, could maintain protocol compliance, and lived within 60 minutes of the study site were eligible for recruitment. Exclusion criteria included specific medical conditions affecting volume status and urine concentration (e.g., heart failure, chronic kidney disease, syndrome of inappropriate antidiuretic hormone, diabetes insipidus, and loop diuretics).

Three patients were enrolled after obtaining informed consent; efforts to recruit more patients were limited due to the uncommon nature of this diagnosis and co-occurrence of renal dysfunction, excluding three patients. The underlying immunocompromising conditions included one patient with idiopathic CD4 lymphocytopenia and two patients with rheumatoid arthritis on anti-tumor necrosis factor therapy. Each participant had two study visits approximately 2 weeks apart. For each visit, they fasted overnight and collected a pre-hydration urine sample upon awakening on the study visit day. A serum pre-hydration sample was collected on-site. Utilizing an oral hydration goal similar to a previous study evaluating hydration and urine pregnancy testing, participants drank 1 L of fluid within 30 minutes, and post-hydration samples were collected after another 60 minutes (5). UHAg and SHAg levels were measured using the MVista Immunoassay. Urine and serum osmolality were also measured. Statistical analyses utilizing a generalized linear model, individual patient, day of testing, and hydration state were modeled as predictors of UHAg, SHAg, urine osmolality, and serum osmolality along with their interaction terms. Analyses utilized SAS software (version 9.4, SAS Institute) with alpha = 0.05.

**Fig 1:**
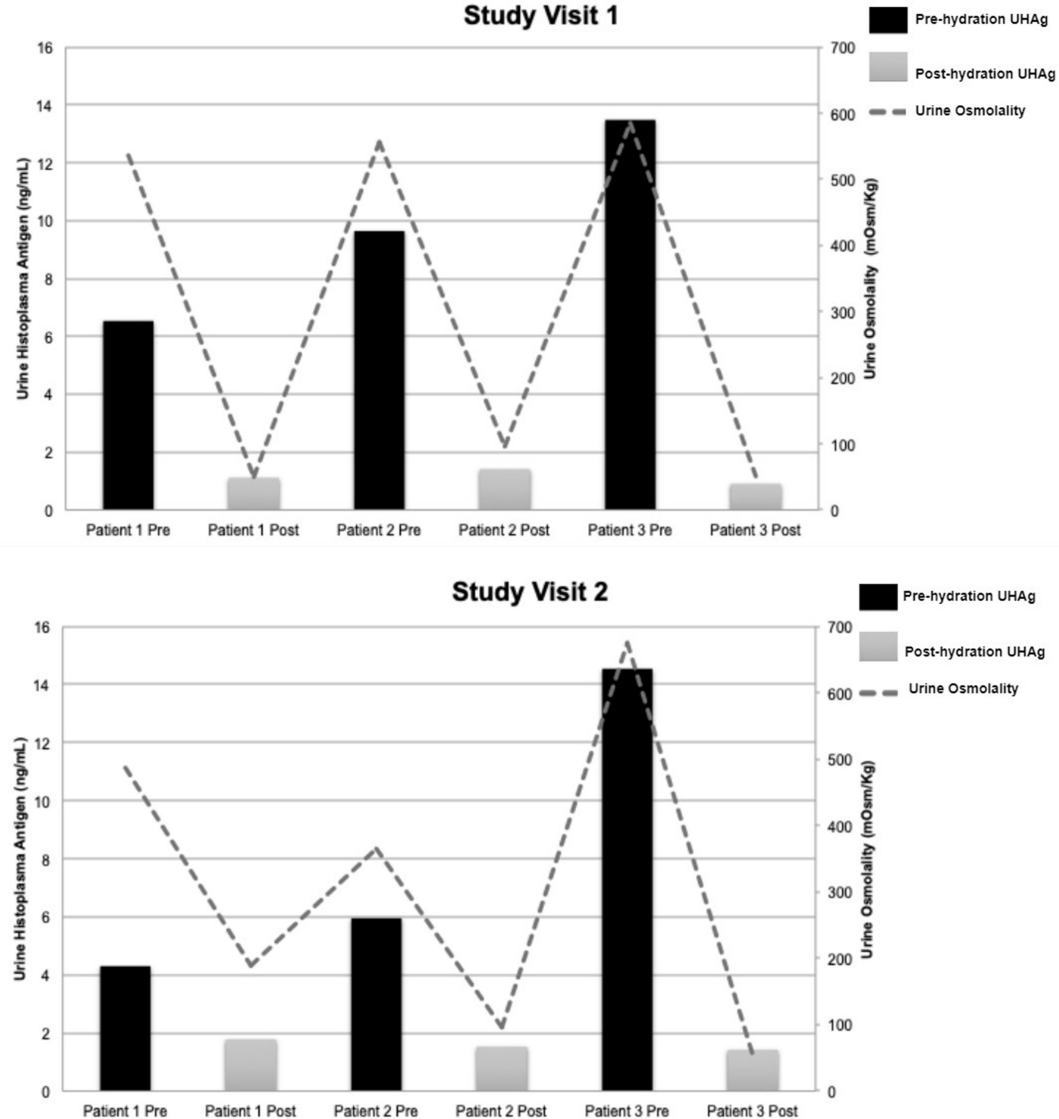
Variations of UHAg levels and urine osmolality with hydration status (Pre = pre-hydration, Post = post-hydration) across study visit 1 (A) and study visit 2 (B).

The mean UHAg pre-hydration level was 9.07 ± 1.88 ng/mL with a mean post-hydration level of 1.36 ± 1.88 ng/mL (*P* = 0.11) (Fig. 1A and B). One patient demonstrated a considerable reduction of 12.85 ng/mL with hydration (pre-hydration 14.01 ng/mL, post-hydration 1.16 ng/mL). While UHAg differences were noted, they did not reach statistical significance. Urine osmolality demonstrated significant differences between mean pre-hydration and post-hydration (534 ± 33 mOsm/kg and 89 ± 33 mOsm/kg, respectively; *P* = 0.02), indicating the oral intake protocol was adequate to physiologically dilute the urine.

Patient 2 was noted to have differences in UHAg pre-hydration levels between study visit 1 (9.64 ng/mL) and study visit 2 (5.95 ng/mL). Of note, pre-hydration urine osmolality was measured at 556 mOsm/kg during study visit 1 and 365 mOsm/kg during study visit 2. Based on these observations, the difference in UHAg may be related to changes in baseline hydration status of patient 2 at the two study visits.

The SHAg levels did not vary significantly with hydration. The mean pre-hydration SHAg level was 2.38 ± 0.20 ng/mL while post-hydration SHAg level was 2.40 ± 0.20 ng/mL (*P* = 0.93). Mean serum osmolality was noted to be 294 ± 1.17 mOsm/kg pre-hydration and 291 ± 1.17 mOsm/pg post-hydration (*P* = 0.11).

This study highlights the impact of hydration on UHAg levels in patients with disseminated histoplasmosis and should be noted while interpreting results during initial diagnosis and in follow-up. While UHAg differences were not statistically significant, we believe they are clinically relevant, and the lack of statistical significance was related to small study size. When patients with a lower burden of disease are well hydrated, it is plausible that false-negative UHAg results may occur. Furthermore, some patients may have a high enough burden of disease that SHAg is positive while dilute urine may yield false-negative UHAg results. This may explain prior discordant results noted between UHAg and SHAg (6). Hydration status may be a factor that explains variability when UHAg levels are monitored over time as has been hypothesized in previous studies (1–4).

Based on these observations, collection of the first morning urine or the most concentrated sample could be considered to potentially lessen the variability of UHAg although as noted in patient 2, variability may still occur. Additionally, collecting both UHAg and SHAg may improve testing results although future larger studies are needed to support both of these suggestions and to evaluate whether the observed variability of UHAg noted is statistically significant.
